# Corbi: a new R package for biological network alignment and querying

**DOI:** 10.1186/1752-0509-7-S2-S6

**Published:** 2013-10-14

**Authors:** Qiang Huang, Ling-Yun Wu, Xiang-Sun Zhang

**Affiliations:** 1 National Center for Mathematics and Interdisciplinary Sciences, CAS, Beijing, China 100190; 2 Institute of Applied Mathematics, Academy of Mathematics and Systems Science, CAS, Beijing, China 100190

## Abstract

In the last decade, plenty of biological networks are built from the large scale experimental data produced by the rapidly developing high-throughput techniques as well as literature and other sources. But the huge amount of network data have not been fully utilized due to the limited biological network analysis tools. As a basic and essential bioinformatics method, biological network alignment and querying have been applied in many fields such as predicting new protein-protein interactions (PPI). Although many algorithms were published, the network alignment and querying problems are not solved satisfactorily. In this paper, we extended CNetQ, a novel network querying method based on the conditional random fields model, to solve network alignment problem, by adopting an iterative bi-directional mapping strategy. The new method, called CNetA, was compared with other four methods on fifty simulated and three real PPI network alignment instances by using four structural and five biological measures. The computational experiments on the simulated data, which were generated from a biological network evolutionary model to validate the effectiveness of network alignment methods, show that CNetA gets the best accuracy in terms of both nodes and networks. For the real data, larger biological conserved subnetworks and larger connected subnetworks were identified, compared with the structural-dominated methods and the biological-dominated methods, respectively, which suggests that CNetA can better balances the biological and structural similarities. Further, CNetQ and CNetA have been implemented in a new R package Corbi (http://doc.aporc.org/wiki/Corbi), and freely accessible and easy used web services for CNetQ and CNetA have also been constructed based on the R package. The simulated and real datasets used in this paper are available for downloading at http://doc.aporc.org/wiki/CNetA/.

## Introduction

In the systems biology era, more and more biologists focus on the biological systems instead of individual molecules. The biological networks such as protein-protein interaction (PPI) networks are the most natural and efficient approaches for studying and modeling the complex biological systems. In the last decade, plenty of biological networks are built from the large scale experimental data produced by high-throughput techniques as well as literature and other sources [1-7]. However, the huge amount of network data have not been fully utilized due to the limited biological network analysis tools. One of the basic and important problems in the fields of biological network analysis is network alignment. In brief, network alignment aims to identify biological conserved subnetworks among two or more different biological networks and evaluate the global or local similarity of biological networks. A simplified version of network alignment is equivalent to the maximum subgraph isomorphism problem, which is NP-complete, i.e. there is no efficient algorithm according to the computational complexity theory. The biological network alignment may be much harder due to the complex evolutionary events such as gene mutations and duplications.

There are many network alignment algorithms published in literature, e.g., Græmlin [8], MRF based method [9], MNAligner [10], IsoRank [11,12], IsoRankN [13], MI-GRAAL [14]. The network alignment problem is often formulated into an optimization problem, and solved by heuristic algorithms such as the seed extend algorithm [14]. The network alignment is essentially a multi-objective optimization problem, and there are two objectives: the biological similarity and the structural similarity. Based on the different trade off strategies between two objectives, we categorized the network alignment methods into three groups: structural-dominated, biological-dominated, and balanced. Due to the large sizes of biological networks, the computational complexity becomes the most important issue for the network alignment methods. New models that can appropriately balance the biological and structural similarities and algorithms that can efficiently and effectively solve the large scale problem are extremely demanded in the fields of systems biology.

Biological network alignment methods are usually designed specific to different variants of the problem. Fast algorithms are developed for network querying problem, that is, given a small and simple network, identifying the best matched subnetworks in a large and complex network. Pairwise and multiple large network alignment problems are also separately considered because of the computational complexity and the desired mapping types. The one-to-one mapping is often used for pairwise network alignment to find the precise matching, while many-to-many mapping is more used in multiple network alignment for studying the evolutional events. In a previous work [15], we developed a network querying method named CNetQ. By formulating the network querying problem as a conditional random fields (CRF) model, which is widely used in the fields of machine learning, we designed efficient algorithms specific to different structures of query networks. In this paper, by adopting a novel iterative bi-directional mapping strategy, we further extended CNetQ to the pairwise network alignment problem with one-to-one mapping, and named the new method CNetA. In this study, four structural and five biological measures were utilized to assess the performance of network alignment methods. CNetA was compared with a seed-and-extend method MI-GRAAL [14], and two simple BLAST-based methods [16] as well as CNetQ. The computational experiments on the simulated and real data showed that CNetA can properly balance the biological and structural similarities and achieve more accurate results in terms of both nodes and networks.

## Methods

### Network alignment problem

**Figure 1:**
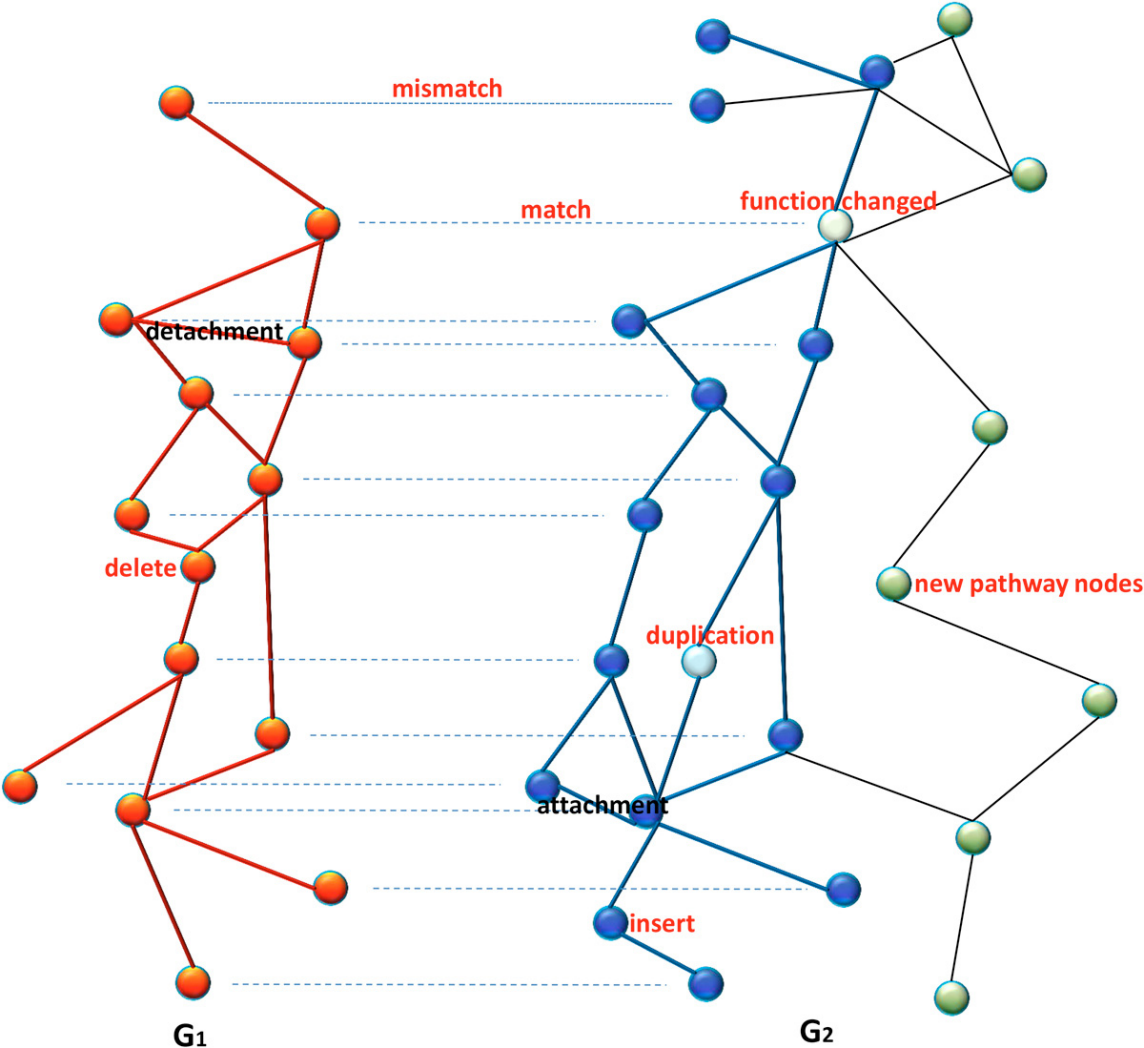
**An example of network alignment between *G*_1 _and *G*_2_**. A network alignment model needs to deal with the node mutations (e.g., insertion, deletion, duplication, mismatch, and also function changed) and the edge mutations (e.g., detachment, attachment).

A biological network is often mathematically defined as a graph *G *= (*V, E*), where *V *is the set of nodes (e.g. proteins) and *E *is the set of edges (e.g. interactions between proteins). The edge can be directed or undirected. For example, PPI networks are usually modeled as an undirected graphs, and metabolic networks and gene regulatory networks are usually regarded as directed graphs. Using the graph representations, network alignment problem can be generally described as follows. Given two biological networks *G *= (*V, E*) and *G*' = (*V*', *E*'), network alignment aims to find the maximum conserved subnetworks of *G *and *G*'. The word "conserved" means that the corresponding nodes of two subnetworks are biologically similar and the structures of two subnetworks are also similar. As illustrated by a simple example in Figure 1, the network alignment should consider the evolutional events such as node insertion/deletion and edge attachment/detachment.

### Conditional random field model

We developed a conditional random fields model CNetQ to address the network querying problem in [15], which is a special case of network alignment. Given a small network (query) and a large network (target), network querying is to find the subnetworks of target similar to query. The CNetQ model is briefly described as follows and readers can refer to [15] for more details. Suppose a network *G *= (*V, E*) is queried in a target network *G*' = (*V*', *E*'). If we consider *G*' as a label set, that is, *V*' are all possible labels and *E*' are the relations between labels, the network querying problem can be relaxed to a labeling problem, that is, to find the best label from *G*' for each node in *G*. We defined a CRF model to assign a conditional probability to each possible labeling solution *Y *⊆ *G*':

$$\begin{array}{llll} \text{Pr(}Y\text{|}G\text{)}= & \frac{1}{Z\left(G\right)}\underset{v_{i}\in V}{\prod }f_{N}\left(y_{i},G,i\right)\; & & \;\\ & \underset{\left(v_{i},v_{j}\right)\;\in E}{\prod }f_{E}\left(y_{i},y_{j},G,i,j\right)\; & & \;\\ & \; & \end{array}$$

where *y_i _*is the label in *Y *assigned to node *vi *in *G*, *f_N _*, *f_E _*are called node and edge feature functions respectively, and *Z*(*G*) is the normalization factor. The solution with the maximum conditional probability is extracted as the final result. Considering the node insertions/deletions, we use the following definitions for node and edge feature functions respectively [15]:

$$\begin{array}{c} f_{N}\left(y_{i},G,i\right)=S\left(v_{i},y_{i}\right),\\ f_{E}\left(y_{i},y_{j},G,i,j\right)=\frac{S\left(v_{i},y_{i}\right)+S\left(v_{j},y_{j}\right)}{2}W\left(y_{i},y_{j}\right). \end{array}$$

where *S*(*v_i_, y_i_*) and *W *(*y_i_, y_j _*) are the non-negative node and edge similarity scores respectively. The details and discussion about feature functions can be found in [15,17].

### Iterative bi-directional mapping strategy

There are two key issues when applying CNetQ to the pairwise network alignment problem. The first one is that CNetQ allows many-to-one mapping while pairwise biological network alignment often requires one-to-one mapping. For network querying, it is very rare that two nodes in the query network are matched to one node since the query is very small. The many-to-one mapping becomes common while the size of query network increases. The gene duplications may result in many functional similar proteins. The other issue is the asymmetric results. That is, the result of querying *X *in *Y *is different from that of querying *Y *in *X*, which is not expected in pairwise biological alignment.

In order to overcome these issues, we proposed a novel network alignment method CNetA by using an iterative bi-directional mapping strategy. At each iteration, we do twice network querying by applying CNetQ, that is, querying *G *in *G*' and vise versa, and fix the common matching pairs of two results in the rest iterations and update the node feature function. The process is repeated until there is no new common matching pairs. More details of the iterative bi-directional mapping strategy can be found in [17].

### Evaluation measures

For the simulated data, network alignment results are compared with the true alignments. But for the real biological data, the true alignments are not available. We collected and improved several evaluation measures in literature to assess the performance of network alignment methods. In detail, we used four structural measures, including that the number of matching pairs (MP), edge correctness (EC) [14], edge accumulated coverage (EAC), the size of largest common connected subgraph (LCCS) [14], and five biological measures, that is, the fraction of matching pairs that share GO terms (SGO) [14], GO coverage, the number of hit pathways(HP) [8], pathway average coverage (PAC) [8], KEGG orthologous proteins (OP) [8]. More details and discussion of evaluation measures can be found in [17].

### Simulated data

In the preliminary version of this paper presented in the conference IEEE ISB 2012 [17], we only test the new method on the real PPI data. Since the true alignment of the real data is not unknown, it is difficult to precisely and directly assess the performance of network alignment methods. Therefore, in this paper, we addressed this issue by generating the simulated network data in view of a simple gene duplication event model [18]. We assume that 1) the duplication of a gene induces the duplication of relevant protein and interactions, 2) the duplicated and the original proteins tend to interaction loss since the redundancy, 3) the interaction loss in duplication events are more probable than randomly interaction loss, 4) the duplications will not occur on the duplicated nodes, 5) the duplications will not happen twice on the same node or on two adjacent nodes (to avoid the correlation of two duplication events).

Based on the above assumptions, given a network *G *= (*V, E*) and the maximum number of duplications *N *, the duplicated network *G*' was generated as follows:

1. Set the duplication number *n *= 0, the duplicated node set *D *= ∅, the adjacent node set *A *= ∅, the remaining node set *R *= *V*.

2. Randomly select a node *v *in *R *and generate a duplicated node *v*' with the same interaction pattern as *v *in *G*.

3. Simulate interaction loss for duplication event. Randomly remove a fraction *p*1 of edges for *v*' and a fraction *p*2 of edges for *v*, where *p*1 ≥ *p*_2_.

4. Set *n *= *n *+ 1, *D *= *D *∪ {*v*}, *A *= *A *∪ *N *(*v*), *R *= *R \ *({*v*} ∪ *N *(*v*)), where *N *(*v*) is the neighboring nodes of *v *in *G*.

5. Repeat step 2-4 until *n *≥ *N *or *R *is empty.

6. Simulate randomly interaction loss. Randomly remove a small fraction *p*3 of edges, where *p*1 ≥ *p*2 ≫ *p*_3_.

The duplicated nodes were assigned the same sequences of their original nodes respectively, to avoid the effect of alignment for non-duplicated nodes as much as possible. For more complicated model of evolutionary gene duplication events, the probabilities of lost and gained interactions will be related with the network structure. Since our goal is to evaluate the network alignment methods, how to set up a reasonable duplication model is beyond the scope of this paper.

## Results

### Comparison settings

The new method CNetA was compared with four other methods to evaluate its performance. The first method MI-GRAAL [14] is structural-dominated and combines four structural similarities and one sequence similarity. The second one is biological-dominated method named BLASTQ, which only use the sequence similarity based on BLAST [16]. BLASTQ simply queries each node of *G *in *G*' by finding the best BLAST hits. The third method is BLASTA which further integrates BLASTQ with the same iterative bi-directional mapping strategy in CNetA. The last one is the network querying method CNetQ [15]. Note that BLASTQ and CNetQ usually do not guarantee one-to-one mapping for large network alignments. We only extracted the one-to-one matching pairs as the final results of CNetQ and BLASTQ. As discussed in [17], we used the same parameters for CNetA and CNetQ.

### Results on simulated data

In this section, we compared the network alignment results on the simulated network data. Since the true alignments for simulated data are known, we evaluated the alignment results by two types of accuracy which are computed as the fractions of correctly aligned nodes in duplicated nodes and all nodes respectively, and the structural measures. For the node that is duplicated, it is regarded as correctly aligned when the original node is aligned with the corresponding one and the new node is aligned with gap.

The original network *G *used for generating simulated data is the real yeast PPI network [19], which contains 2390 proteins and 16127 interactions. The sequences of yeast proteins were downloaded from Saccharomyces Genome Database (SGD, http://www.yeastgenome.org) [20]. The parameters for simulation are set as follows. *N *and *p*_3 _are fixed as 100, 0.005, respectively. To investigate the effect of the parameters *p*_1 _and *p*_2_, we tried five different settings, i.e., (0.5, 0.2), (0.4, 0.2), (0.2, 0.1), (0.2, 0.05), (0.1, 0.05). Given a network *G *and a pair values of (*p*_1_, *p*_2_), we randomly generated 10 simulated networks and their true alignments with *G *respectively. Therefore, there are totally fifty simulated network datasets.

To find the best combination of similarities, MI-GRAAL need to run 31 times for each pair of networks, which is very time-consuming. Therefore, in this study, we use all the five similarities for fifty simulated network datasets. Moreover, the software downloaded from the MI-GRAAL website can not run successfully for some datasets. Only the successful alignment results are shown and analyzed in this section.

**Figure 2:**
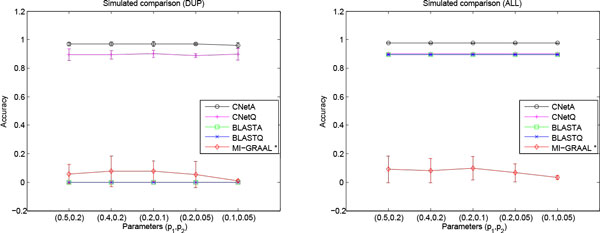
**The simulated experimental results based on yeast PPI network**. Figure 2-1, the accuracy for duplicated proteins (DUP). Figure 2-2, the accuracy for all proteins (ALL). * means that the results for MI-GRAAL are only a part of the simulated datasets since that MI-GRAAL method failed on several simulated datasets.

The results of five methods are compared in Table 1 and Figure 2. Since the results are robust for different parameter settings of (*p*_1_, *p*_2_), the results in Table 1 are the mean values in all datasets. More details of the simulated data results can be found in Additional file 1.

**Table 1:** Simulated comparison

Method	MI-GRAAL	CNetQ	CNetA	BLASTQ	BLASTA
MP	2390	2155	2385	2133	2133

EC	38.00%	88.60%	99.47%	72.33%	72.33%

LCCS	1559(6013)	1761(14001)	1989(15737)	1607(11301)	1607(11301)

Accuracy DUP	8.33%	89.57%	96.71%	0	0

Accuracy ALL	7.78%	90.20%	97.57%	89.40%	89.40%

Figure 2 shows that CRF based methods reveal correct alignments for most duplicated nodes as well as non-duplicated nodes. The accuracy of MI-GRAAL is very low for both types of nodes. The BLAST based methods completely failed for duplicated nodes but succeeds for non-duplicated nodes. All methods show robust performance in different parameter settings.

MI-GRAAL obtains low accuracy for the duplicated nodes due to that the duplicated nodes are mainly biological similar instead of structural similar and MI-GRAAL is dominated by the structural information. On the other hand, the BLAST based methods are completely failed for duplicated nodes since they can not distinguish the original nodes and duplicated nodes, and produce many-to-one mappings. The CRF based methods achieve the best results, which shows that the edge feature function used in CRF based methods is helpful for utilizing the structural information to discriminate the biological similar nodes.

It is unexpected that MI-GRAAL fails to align the non-duplicated nodes which have both high structural and biological similarities. One of the possible reasons is that MI-GRAAL is mislead by the network structure subtly disturbed by the duplicated nodes and edges. In other words, the structural-dominated methods such as MI-GRAAL may be too sensitive to the network structural changes because they do not fully exploit the biological similarity, and may be not good at identifying biological similar nodes with a few changes of network linkages. The BLAST based methods achieve high accuracy on the non-duplicated nodes since the sequences of nodes are not changed. The CRF based methods acquire the best accuracy again, where the node feature function makes much contribution.

As show in Table 1, CNetA gets the best results except MP is slightly smaller than that of MI-GRAAL. Similar with the majority vote, MI-GRAAL ignores the node sequence similarity and overemphasizes the structural similarities. Although it matches every node in the smaller network, it seriously overlooks the node information and incorrectly align many nodes. On the other hand, BLAST based methods do not utilize the structural information and can not distinguish the true alignment from biological similar nodes. The computational experiments show that the balance between the structural and biological similarities are very important for the biological network alignment. CRF based methods fully exploit both structural and biological information and obtain a good balance between them. Furthermore, the accuracy of CNetA is significantly improved over CNetQ which validates the effectiveness of the iterative bi-directional mapping strategy.

### Results on real data

We further compared the network alignment methods on three real PPI network pairs, which were initially used in [14]. The related evaluation measures were computed by using Matlab Bioinformatics Toolbox. Three GO domains biological process, cellular component, and molecular function are abbreviated as BP, CC, MF respectively.

MI-GRAAL [14] does not produce same alignment in each run since it has a random seed procedure. The alignment results also depend on the combinations of similarities. For simplicity, we adopted the most stable combinations of similarities provided in [14]. For each instance, the alignment with the maximum EC in five runs was chosen as its final result.

#### Yeast-Human PPI network alignment

**Figure 3:**
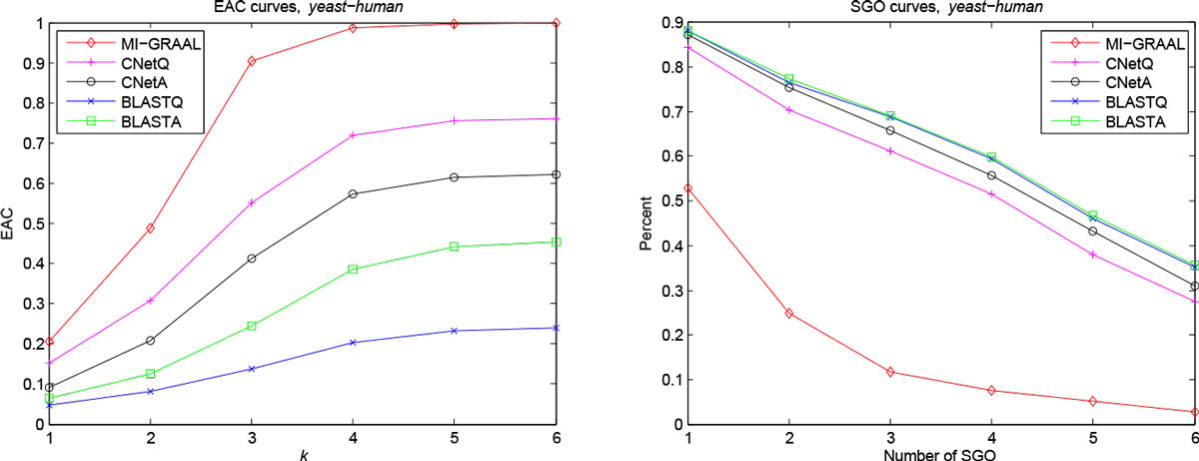
**EAC and SGO curves for comparing *yeast *and *human *PPI networks**. Fig 3-1, EAC curves. The x axis is the allowable maximum distance *k *between two nodes matched to the end nodes of an edge. The y axis is *EAC*(*k*). The legend indicates the compared methods. Fig 3-2, SGO curves. The x axis is the number of SGO. The y axis is the percent of matching protein pairs.

The same Yeast PPI network and sequence data in simulated data, and the human PPI network and sequences from [21] were used in the first experiment. Table 2 and Figure 3 summarized the results of five methods.

**Table 2:** Yeast-human alignment results

Method	MI-GRAAL	CNetQ	CNetA	BLASTQ	BLASTA
MP	2390	1029	1694	1297	1672

EC	20.52%	15.29%	9.25%	4.81%	6.52%

LCCS	1870(2940)	205(956)	116(376)	47(141)	55(172)

GO coverage (depth ≥ 3)					

MF	12.70%	47.78%	54.61%	55.07%	56.43%
BP	9.91%	52.01%	53.97%	58.55%	58.10%
CC	44.17%	72.20%	72.73%	76.33%	74.74%

KEGG analysis					

OP	24	331	556	583	719

HP	9	26	27	27	27

PAC	9.961%	21.80%	32.35%	31.66%	35.06%

MI-GRAAL reveals the largest structural common subnetwork (LCCS equals to 1870) due to it compels that the result is a complete matching for the smaller network. However, the biological similarity of matching nodes is not so good. As shown in Figure, less than 55% and 15% matching pairs share at least one or three common GO terms respectively, while the fractions of other methods are larger than 80% and 60%. MI-GRAAL performs poor in other biological measures, too. For example, only 9 pathways from total 30 KEGG pathways with the same definition in two species are hit with PAC 9.961%, while other methods hit at least 26 pathways with PAC larger than 20%. On the other side, BLASTQ and BLASTA outperform other methods in terms of all biological measures, whereas, with the worst scores of structural measures. CNetQ and CNetA achieve better trade-off between the biological and structural similarity. They find the alignments with much higher biological similarity at the cost of slightly lower structural similarity than MI-GRAAL, while the biological similarity scores are even comparable to the BLAST based methods.

The power of iterative bi-directional mapping strategy is exhibited by the improvements of BLASTA over BLASTQ in almost all the measures except the comparable GO coverage. Similarly, CNetA hits one more pathway with larger PAC and more orthologous protein pairs than CNetQ. CNetA also finds more functional conserved matching pairs based on GO coverage and SGO. These results indicate that the iterative bi-directional mapping strategy is not only useful to identify one-to-one mappings, but also helpful to reveal more functional conserved alignments.

#### Campylobacter jejuni-Escherichia coli PPI network alignment

**Figure 4:**
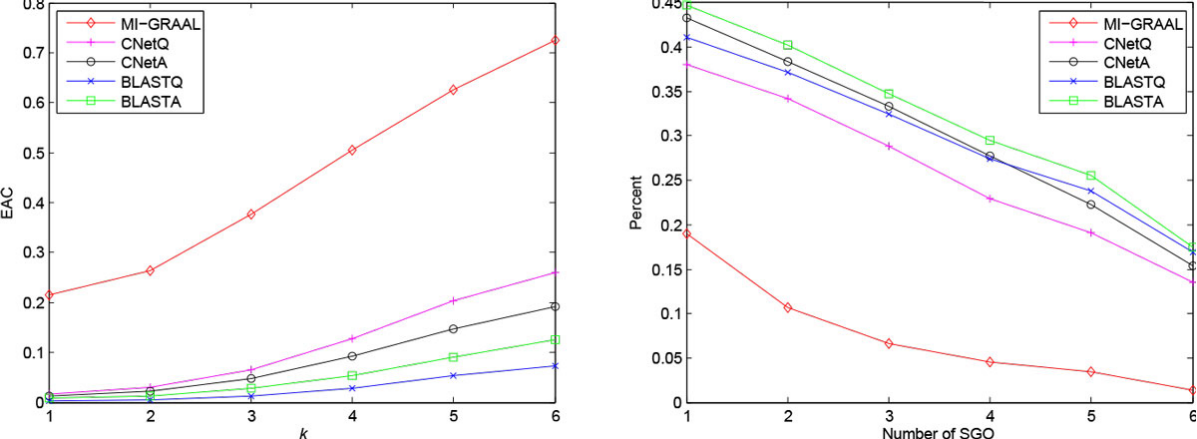
**EAC and SGO curves for comparing *C. jejuni *and *E. coli *PPI networks**. The legends are the same as Figure 3.

Next, *C. jejuni *PPI network with 1091 proteins and 2966 interactions [22], and *E. coli *PPI network with 1873 proteins and 3803 interactions [23] were aligned by five methods. The network data are slightly different from [14]. Table 3 and Figure 4 show the results of five methods.

**Table 3:** C. jejuni-E. coli alignment results

Method	MI-GRAAL	CNetQ	CNetA	BLASTQ	BLASTA
MP	1091	444	677	533	711

EC	21.48%	1.69%	1.21%	0.37%	0.84%

LCCS	513(544)	7(6)	7(6)	3(2)	4(3)

GO coverage (depth ≥ 3)					

MF	6.94%	27.70%	30.58%	30.96%	32.21%
BP	4.32%	23.87%	26.44%	28.33%	30.38%
CC	5.44%	12.39%	14.33%	13.88%	14.35%

KEGG analysis					

OP	11	95	146	152	206

HP	4	10	11	10	11

PAC	10.23%	15.40%	29.61%	21.91%	36.68%

The performance of five methods is similar to that in the first experiment. CNetA and BLASTA hit 11 pathways with PAC larger than 29% in total 12 KEGG pathways. CNetQ and BLASTQ are a little worse, while MI-GRAAL gives the worst scores. Again, CNetA and BLASTA get significant improvement over CNetQ and BLASTQ respectively. We note that CNetA gets much lower structural scores than MI-GRAAL. It is possibly because that the PPI networks are not complete and consist of many disconnected subnetworks.

#### Mesorhizobium-Synechocystis PPI network alignment

**Figure 5:**
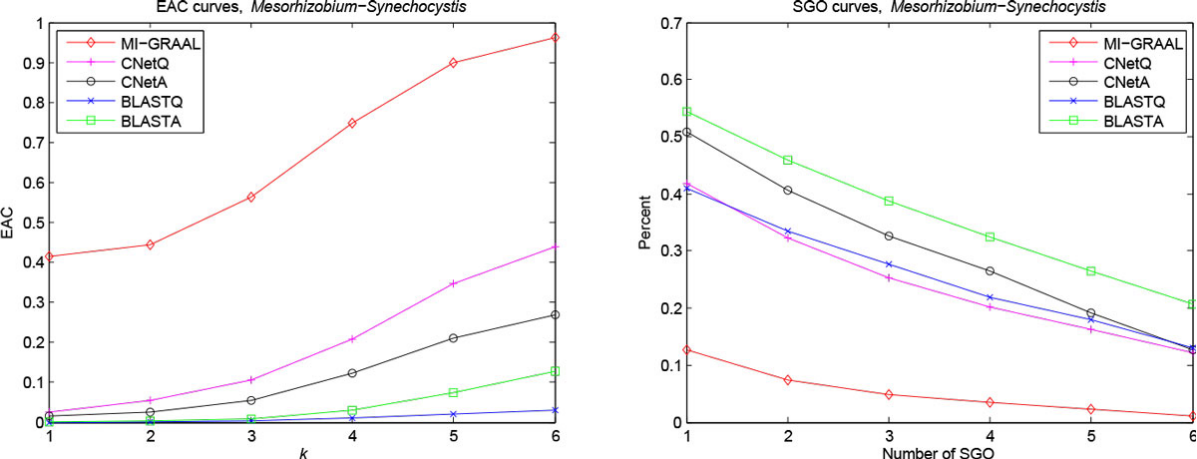
**EAC and SGO curves for comparing *Mesorhizobium *and *Synechocystis *PPI networks**. The legends are the same as Figure 3.

The last experiment, we aligned *Mesorhizobium loti *PPI network with 1804 proteins and 3094 interactions [24] to *Synechocystis sp. PCC6803 *PPI network with 1920 proteins and 3102 interactions [25]. Table 4 and Figure 5 give the results of five methods, which is similar to the above experiments. Due to lack of adequate annotated orthologous proteins in KEGG database, OP are not calculated for all methods. The low structural scores of CNetA may caused by the incomplete and noisy network data of *Mesorhizobium *and *Synechocystis*.

**Table 4:** Mesorhizobium-Synechocystis alignment results

Method	MI-GRAAL	CNetQ	CNetA	BLASTQ	BLASTA
MP	1803	414	744	414	764

EC	41.37%	2.52%	1.55%	0%	0.097%

LCCS	1153(1158)	31(35)	10(9)	1(0)	2(1)

GO coverage (depth ≥ 3)					

MF	5.67%	26.52%	33.60%	28.16%	38.24%
BP	3.97%	23.84%	24.56%	24.76%	31.67%
CC	1.70%	8.52%	8.23%	9.22%	9.72%

KEGG analysis					

HP	1	1	2	1	2

PAC	1.76%	1.06%	3.43%	0.82%	5.45%

**Note: **The three real experiment results of MI-GRAAL method in corresponding Tables and Figures are a little different with the results in our previous paper [17] since that we found a bug in the MI-GRAAL Runner script file during preparing this paper, fixed it and computed results again.

### Software and web services

We implemented CNetQ and CNetA in a new R package Corbi (http://doc.aporc.org/wiki/Corbi). The latest version of Corbi can be downloaded and installed directly by typing the following command within R: install.packages("Corbi", repos="http://R-Forge.R-project.org")

There are two functions *net.align *and *net.query *for network alignment and querying respectively. A simple usage of two methods is as follows:

library(Corbi)

net.align(net1, net2, nodesim)

net.query(querynet, targetnet, nodesim)

where *net1*, *net2*, *querynet *and *targetnet *are the filenames of the network files and *nodesim *is the filename of the node similarity file. The parameters of two methods can also be tuned through the optional arguments of two functions. More details about the usage of two methods and the format of input files can be found in the help documentation of Corbi.

**Figure 6:**
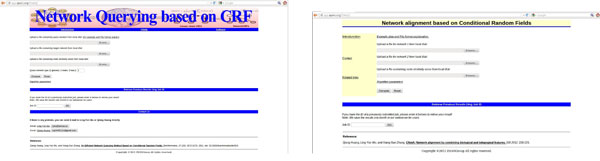
**The interface of web server for CNetQ and CNetA**. Fig 6-1, the interface of CNetQ. Fig 6-2, the interface of CNetA. CNetQ and CNetA are designed as public tools for biological network querying and alignment. Users can upload the networks in an edge based format and the node similarity file directly. The web server will give an alignment result file in a text file and also support downloading the result by job ID later.

As shown in Figure 6, freely accessible and easy used web services for CNetQ and CNetA have also been constructed at http://app.aporc.org/CNetQ/ and http://app.aporc.org/CNetA/, respectively. The web services are implemented based on the R package Corbi. The input for the web services includes two network files and one node similarity file. The alignment results will be shown and can be downloaded from the web server. For each submitted alignment job, the server will assign a job ID. Users can download their results later by the job IDs instead of waiting the program finishes. The input and result files of three experiments on real data in this paper are provided for downloading from the web server directly as the example data. In our web server, these example data run in less than ten minutes.

## Conclusion and discussion

In this paper, we further extended our network querying method to the network alignment problem. The network querying method based on the conditional random fields model does not consider the many-to-one mapping issue which rarely happens in network querying. However, the many-to-one mapping problem becomes serious in network alignment. In order to guarantee one-to-one mapping, a novel iterative bi-directional mapping strategy was proposed. Moreover, the new strategy improves the biological meanings of alignment results. The CRF based network alignment and querying methods were implemented in R package as well as easily used web services.

Fifty simulated networks were generated from real PPI network for comparing network alignment methods. For the noisy or incomplete networks such as *Mesorhizobium *and *Synechocystis *PPI networks, CNetA got much lower structural scores than MI-GRAAL. However, when we compare these methods on the simulated data, CNetA achieves much better structural scores without loss of the biological accuracy. In other words, CNetA well balances the biological and structural information, and is not disturbed by the noisy or incomplete network data, which is very important for downstream systems biology analysis.

Many methods were developed to address network alignment problem. However, there is no well established benchmarks and measures to evaluate network alignment methods. An important reason is lack of the datasets with known optimal alignments. In this paper, we generated a simulated data from an evolutionary model for which the true alignments are available. This may be a promising approach to establish an ideal benchmark databases for network alignment. On the other hand, when using the real data to evaluate network alignment methods, the data quality and completeness of real biological networks should be considered. For example, as shown in this paper, the structural measures may be biased if the networks are not complete. Finally, since the network alignment is essentially a multiple objectives problem and there is a trade-off between the biological and structural similarities, the measures for evaluating network alignment methods need to be carefully designed and selected.

## Abbreviation

MP: Matching pairs; EC: edge correctness; LCCS: Largest common connected subgraph; MF: Molecular function; BP: Biological process; CC: Cellular component; OP: Orthologous proteins; HP: Hit pathways; PAC: Pathway average coverage. The numbers in LCCS are the number of nodes and edges of LCCS respectively. DUP: duplicated nodes. ALL: all the nodes in the alignment networks.

## Competing interests

The authors declare that they have no competing interests.

## Authors' contributions

QH and LYW designed the study and wrote the manuscript. QH implemented the method, performed the experiments and analyzed the data. All authors contributed to discuss on the method, revised the manuscript and approved the final version.

## Supplementary Material

Additional file 1 **Comparison on simulated data**. Figures for the detail comparison of network alignment methods on the simulated data. For the fifty simulated datasets, we further computed the MP, EC, LCCS for the five given parameter settings of (*p*_1_, *p*_2_) as described in Section "Results on simulated data". Click here for file

## Declarations

The publication of this article is from the National Natural Science Foundation of China (Grant No. 60970091 and 11131009) and Shanghai Key Laboratory of Intelligent Information Processing, China (Grant No. IIPL-2012-004).

This article has been published as part of *BMC Systems Biology* Volume 7 Supplement 2, 2013: Selected articles from The 6th International Conference of Computational Biology. The full contents of the supplement are available online at http://www.biomedcentral.com/bmcsystbiol/supplements/7/S2.
