# Supplementary material for: Corbi: a new R package for biological network alignment and querying
Source: BMC Syst Biol. 2013 Oct 14;7(Suppl 2):S6. doi: 10.1186/1752-0509-7-S2-S6 (PMC3851956; doi:10.1186/1752-0509-7-S2-S6)
Supplement: Additional file 1 — Comparison on simulated data. Figures for the detail comparison of network alignment methods on the simulated data. For the fifty simulated datasets, we further computed the MP, EC, LCCS for the five given parameter settings of (p1, p2) as described in Section "Results on simulated data". [file 1752-0509-7-S2-S6-S1.PDF]

Corbi: A new R package for biological network alignment and querying — Supplementary Materials

Qiang Huang, Ling-Yun Wu, and Xiang-Sun Zhang

National Center for Mathematics and Interdisciplinary Sciences
Academy of Mathematics and Systems Science
Chinese Academy of Sciences, Beijing 100190, China

1 Comparison on simulated data

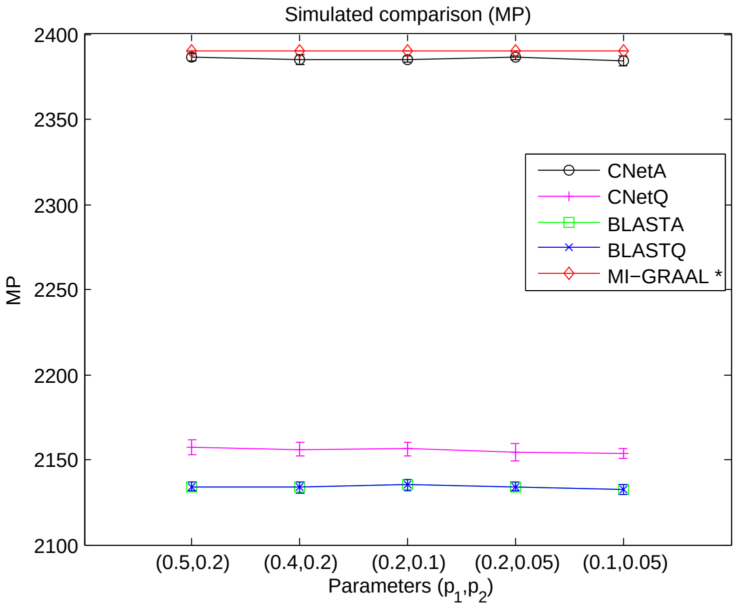

Figure 1: The matching pairs (MP) of all the methods on the simulated data. The x-axis is the parameter setting (p_1, p_2). The y-axis is the number of matching pairs. All the methods have robust results with the different values of (p_1, p_2).

Note: MP: Matching pairs; EC: edge correctness; LCCS: Largest common connected subgraph; DUP: duplicated nodes; ALL: all the nodes in the alignment networks. All BLASTQ curves are the same as BLASTA method since that both methods do not allow the multiple-to-one or one-to-multiple mappings and can not distinguish the duplicated nodes and the equal-sequence-score nodes with only sequence information. * means that the results are only a part of the simulated datasets since that MI-GRAAL failed on other simulated datasets.

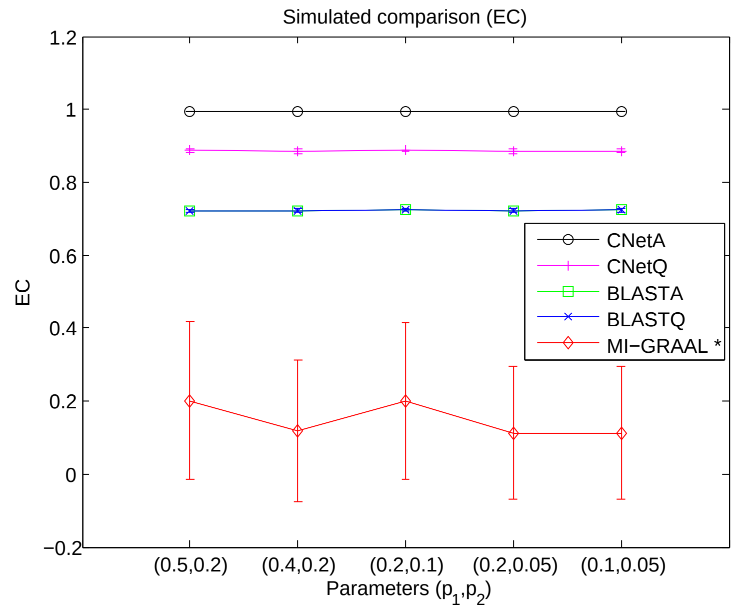

Figure 2: The edge correctness (EC) of all the methods on the simulated data. The x-axis is the parameter setting (p_1, p_2). The y-axis is EC. Here, we do not show the EAC curves for different methods, since that all the methods have high EC values except MI-GRAAL and the EAC curves approximate horizontal lines.

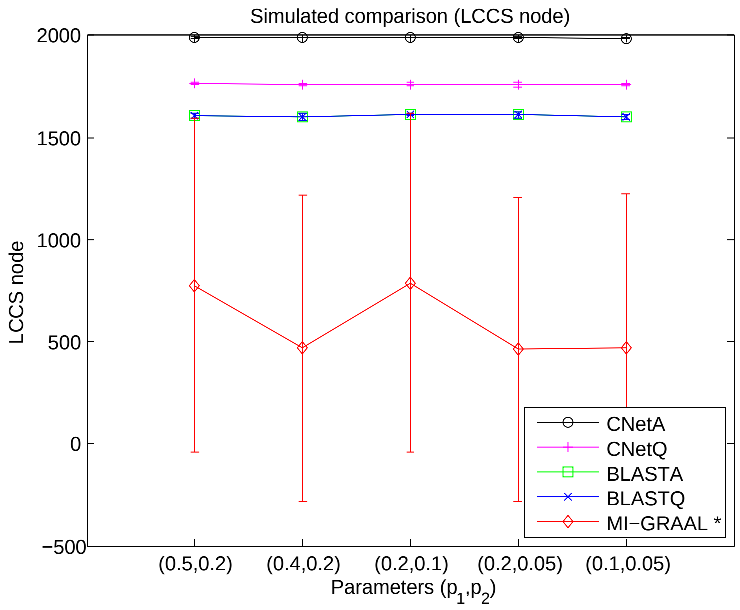

Figure 3: The size of the largest common connected subgraph (LCCS) of all the methods on the simulated data. The x-axis is the parameter setting (p_1, p_2). The y-axis is the size of LCCS.

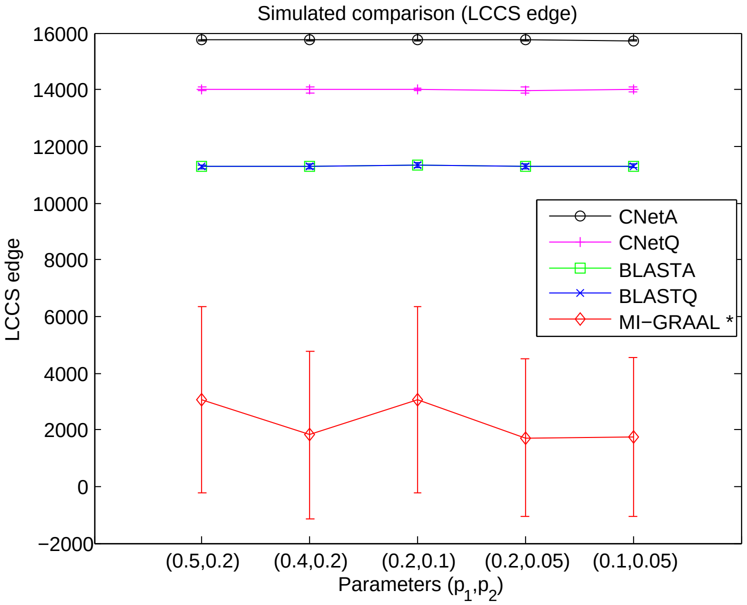

Figure 4: The edge number of the largest common connected subgraph (LCCS) of all the methods on the simulated data. The x-axis is the parameter setting (p_1, p_2). The y-axis is the number of edges in LCCSs.
